# Immature ovarian teratoma in childhood: Case report of successful management of a monster mass in a preschool girl

**DOI:** 10.1016/j.amsu.2022.104147

**Published:** 2022-07-09

**Authors:** Pradeep Kajal, Namita Bhutani, Kirti Saini, Preeti Kadian

**Affiliations:** a Department of Paediatric Surgery, PGIMS Rohtak, Haryana, India; b Deptt. of Pathology, North DMC Medical College and Hindu Rao, Hospital, New Delhi, India; c Deptt. of Gynaecology and Obstetrics, PGIMS Rohtak, Haryana, India

**Keywords:** Case report, Immature, Lump, Ovarian, Teratoma, MRI, Magnetic Resonance Imaging, AFP, Alpha Feto Protein

## Abstract

**Introduction:**

Ovarian teratomas are most common germ cell neoplasms. Immature ovarian teratoma comprises less than 1% of all ovarian teratomas. It usually occurs in first two decades of life.

**Case presentation:**

We report a case of 4 years old female child presenting with pain and huge lump in lower abdomen. On abdominal ultrasonography, it revealed a solid-cystic pelvic lesion arising from left ovary. Magnetic resonance imaging (MRI) corroborated the ultrasonographic findings. She underwent laparotomy with right oophorectomy with excision of the mass. The histopathological examination of the excised mass confirmed it to be immature ovarian teratoma with yolk sac tumor. The patient had an uneventful recovery with no sign of tumor recurrence at a one and a half year follow-up.

**Conclusion:**

In spite of immature ovarian teratomas having aggressive behaviour and lethal outcome, a high degree of suspicion and timely management can translate into a very good eventual prognosis.

## Highlights

• In ovarian mass, patient usually presents with non-specific complaints but a high degree of suspicion and imaging is very important for diagnostic work up.
• Now-a-days tumor markers have also proved to be an important tool for screening the patients of ovarian mass.
• Early diagnosis and management do reduce the risk of complications like torsion, rupture, hydroureteronephrosis and gastrointestinal obstruction.
• It also reduces the probability of metastasis and hence improves event-free and overall survival of the patient.

## 1 Introduction

The word teratoma is derived from a Greek word “teraton” meaning a monster. It is a tumor derived from an abnormal germ cell which undergoes meiotic division. On the basis of histology, ovarian teratomas of two types-mature and immature. The more common of these tumors is mature ovarian teratoma also known as dermoid cyst. Immature ovarian teratoma is uncommon comprising less than 1% of all ovarian teratomas [1,2]. It contains immature tissue derived from three embryologic layers - ectoderm, mesoderm, endoderm. They are typically larger than mature cystic teratoma [3,4]. They usually affect younger age group that is during 1st two decades of life with a peak incidence between 15 and 19 years of age but in our patient, it presented at a much younger age (4 years) which is rare. It mostly presents as a unilateral ovarian mass; still it is common to find benign teratoma in contralateral ovary [5]. These neoplastic lesions differ from mature ovarian teratoma in that they do have malignant behaviour. In view of the rarity and atypical age at presentation in our case, it's being reported with literature review.

## 2 Case presentation

**Fig. 1:**
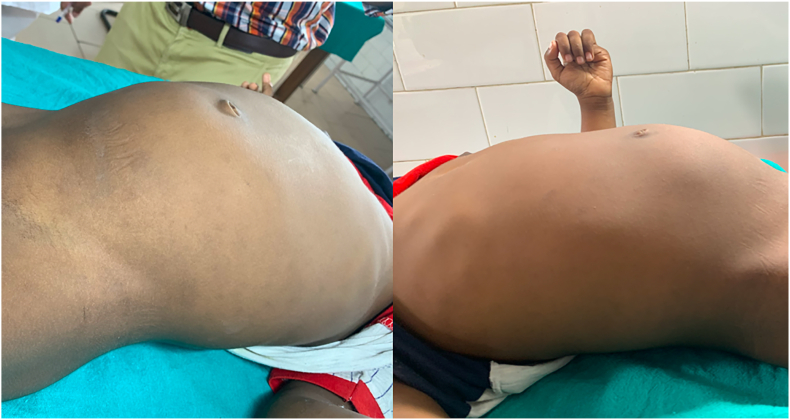
Clinical photograph showing abdominal mass involving pelvic, umbilical, bilateral lumbar and iliac regions.

**Fig. 2:**
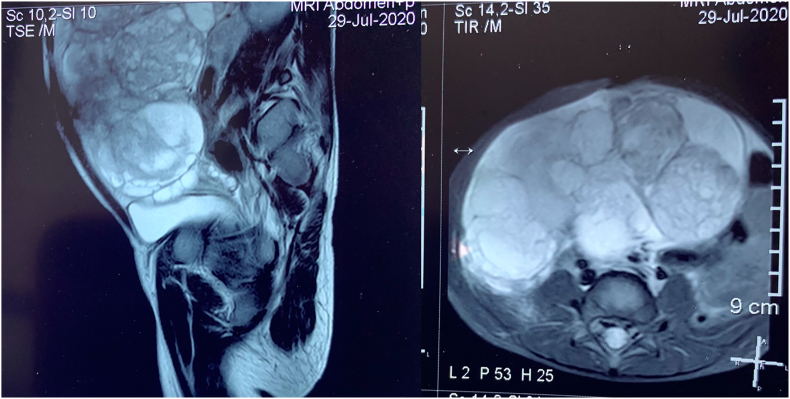
Magnetic resonance imaging showing large lobulated, mixed (solid and cystic) heterogenous signal intensity space occupying lesion in abdomen and pelvis.

**Fig. 3:**
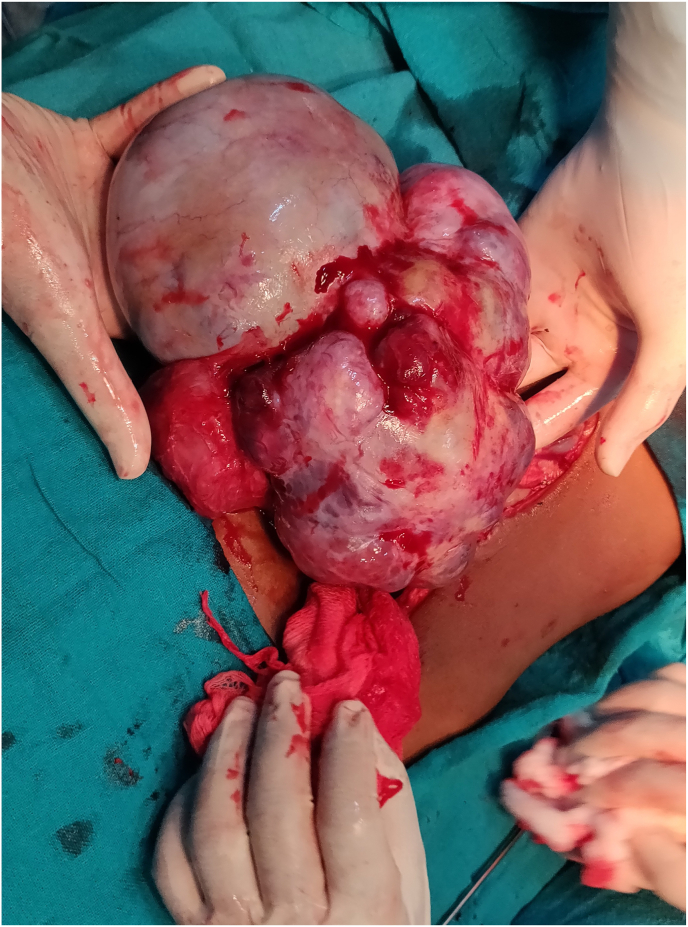
Operative photograph showing the huge mass with a nodular external surface arising from the pelvis.

**Fig. 4:**
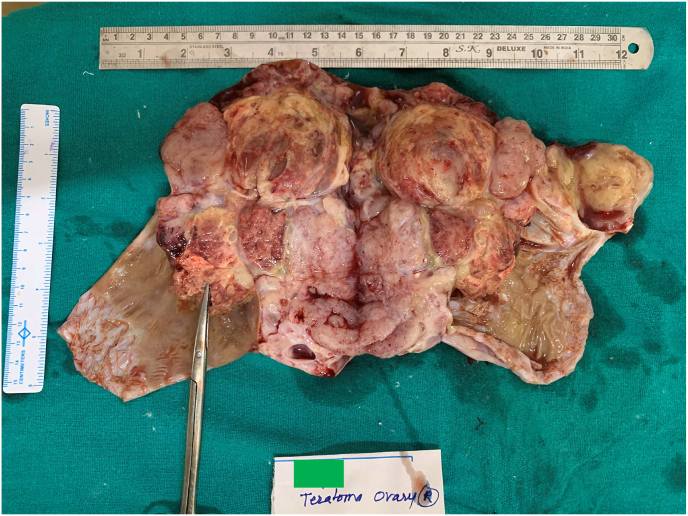
Cut surface of excised specimen show multilobulated grey white necrotic and haemorrhagic areas.

**Fig. 5:**
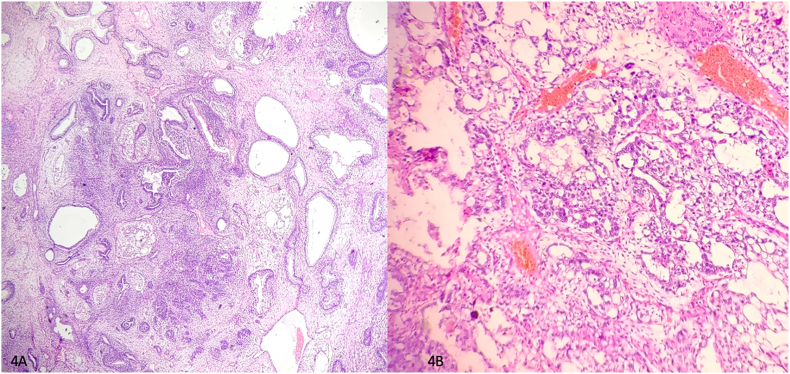
A. Photomicrograph depicting areas of yolk sac differentiation in the tumor (H and E, 100X) B. Photomicrograph showing immature teratoma of ovary; tumor comprising cystic areas along with glandular component (H and E, 40X).

A four-year-old female child from a rural background of Haryana presented to Pediatric Surgery outpatient department with complaints of abdominal lump for previous one month and bleeding per vaginum off and on for 7 days. She had started having pain abdomen for last 4 days as well which was sort of continuous. There was no history of fever or any alteration in bladder or bowel habits. There was no relevant past medical or surgical history. On examination, the patient was afebrile, moderately nourished and stable hemodynamically. Abdominal examination revealed a hard, non-tender, unevenly surfaced lump of about 20*20 cm with indistinct margins present in and involving pelvic, bilateral iliac, umbilical, bilateral lumbar and epigastric quadrants of the abdomen (Fig. 1). It seemed to arise from the pelvis as the lower limit of the lump could not be reached and was movable to some extent in supero-inferior and lateral directions. Systemic examination, all hematological and routine investigations were within normal limits. Abdominal ultrasonography revealed a large, lobulated, solid-cystic, pelvic lesion measuring 20*18 cm extending upwards and involving major part of the abdominal cavity. On abdominal magnetic resonance imaging (MRI), there was a large lobulated, mixed (solid & cystic) heterogenous signal intensity space occupying lesion in abdomen & pelvis measuring 19.2 * 16.0 * 19.2 cm; most likely a teratoma (Fig. 2). The patient was taken up for surgery and was operated upon by the first author who has an experience of 14 years of managing pediatric and neonatal surgical cases in the only government tertiary care centre of the state catering to a large population of patients. On abdominal exploration, there was a large lobulated mass arising from and involving the right ovary and occupying almost the whole abdomen. There was about 50 ml of clear serous fluid in the peritoneal cavity. Bilateral fallopian tubes and uterus were free from any obvious tumor infiltration (Fig. 3). Right oophorectomy with excision of the attached mass along with adjoining segment of the right fallopian tube was done. The cut section of the mass showed multilobulated grey-white necrotic and hemorrhagic areas with some cystic areas as well (Fig. 4). The post-operative period was uneventful. Histopathological examination of the submitted mass confirmed the diagnosis of immature teratoma and yolk sac tumor (Fig. 5). The resected segment of ipsilateral fallopian tube was free from tumor infiltration. Patient was discharged on fourth post-operative day in fair health. She is on a regular 3 monthly follow-up in outpatient department for last one and a half year. She is asked about any new complaint and investigations in the form of complete hemogram, urine examination, liver and kidney function tests, chest X-ray and abdominal ultrasonography are done on each visit. There is no evidence of any tumor recurrence till the last visit 1 month ago.

## 3 Discussion

Overall, immature teratoma is most frequently found in sacrococcygeal region, which happens to be the most common site for all types of teratomas in childhood [6]. Ovary and testis are next most common sites of immature teratoma. Other anatomical sites involved are central nervous system, retroperitoneum, soft tissues of head and stomach. Our case is of right ovarian immature cystic teratoma along with yolk sac tumor. Yolk sac tumor (also known as endodermal sinus tumor) is a malignant primitive germ cell tumor; they are histologically similar to the mesenchyme of primitive yolk sac. Yolk sac tumor can be found in a pure form or mixed with other germ cell tumors [7]. They are commonly found in pediatric age group and can be found anywhere in body.

Immature ovarian teratomas are graded histologically based on amount and degree of cellular maturity: having grade 1 mature and grade 3 immature. For proper grading, multiple sections of primary lesion and wide sampling of peritoneal implants are done. Both primary lesion and implants should be graded according to most immature tissue present [3]. Importance of histological grading is that it is an indicator of prognosis; low grade tumor has good prognosis than high grade tumor. Gonzalez- Crussi grading system is based on amount of immature tissue within tumor having grade 0- mature (benign), grade 1 - (<10% immature) probably benign, grade 2 – (10–50% immature) possibly malignant, grade 3 - (>50% immature) frankly malignant [8].

There are some classical tumor markers for these germ-cell tumors. AFP is useful for diagnosing both yolk sac tumor and immature teratoma. And for discrimination between these two Kawai et al. study states that 94.4% of patients with yolk sac tumor exceeded an AFP level of 1000 ng/ml and in 95.2% cases of immature teratoma it was less than 1000 ng/ml. Other tumor marker used for immature teratoma is CA19-9 with a positivity rate of 57.1% [9]. The role of tumor markers includes: diagnostic screening, evaluation of therapeutic effectiveness and permit earlier detection of recurrent cancer. But in present case, neither AFP nor CA19-9 level was raised. Our diagnosis was made on the basis of histopathological examination. Differential diagnoses of our case are: mature cystic teratoma with microscopic foci of immature elements, mature solid teratoma (similar age distribution and gross appearance, except for absence of necrosis), malignant neuroectodermal tumor (as immature mitotically active neuroectodermal tissue is present in immature teratoma also), malignant mixed mesodermal tumor (as cartilage is present in both tumors).

Treatment for immature ovarian teratoma is grade dependent; that is patients with stage 1 grade 1 immature teratoma are often treated with surgical resection of the mass due to favourable prognosis and very low risk of relapse. In a database study by Pashankar in 2016, out of 179 patients (98 children and 81 adults) he reported that 90 children were treated with surgery alone, and all adults received adjuvant chemotherapy [10]. And grade 2 or 3 or advanced stage teratomas require operative interventions combined with chemotherapy. As our case is of stage 1 grade 1 tumor and no peritoneal implants were seen, so was managed by surgical intervention only. And she is now on regular follow-up with no evidence of tumor recurrence. These tumors do have malignant behaviour but they still have low potential to metastasize depending on their grading [11]. The most reported distant sites of blood-borne metastasis are liver, lungs and brain [12]. According to the data in English literature, prognosis of germ cell tumors depends on age, size, staging and grading of primary tumor and implants.

## 4 Conclusion

In ovarian mass, patient usually presents with non-specific complaints but a high degree of suspicion and imaging is very important for diagnostic work up. Now-a-days tumor markers have also proved to be an important tool for screening the patients of ovarian mass. Early diagnosis and management not only reduce the risk of complications like torsion, rupture, hydroureteronephrosis and gastrointestinal obstruction but also reduces the probability of metastasis and hence improves event-free and overall survival of the patient.

The authors state that the work has been reported in line with the SCARE 2020 criteria [13].

## Ethical Approval

Not Applicable

## Sources of funding

NIL

## Author contributions

1. Pradeep Kajal: Operated upon the patient.

2. Namita Bhutani: Conceptualised the article, did the final editing and submitted the article.

3. Kirti Saini: Reviewed the literature and wrote the article.

4. Preeti Kadian: Arranged the photographs and managed the clinical part.

## Registration of research studies

NOT APPLICABLE FOR CASE REPORTS.

## Consent to publication statement

Written informed consent was obtained from the patient for publication of this case report and accompanying images.

## Guarantor

DR PRADEEP KAJAL.

DR. NAMITA BHUTANI.

## Provenance and peer review

Not commissioned, externally peer-reviewed.

## Declaration of Competing interest

The authors declare that they have no competing interest.
